# Developmental Postural Modeling: A Historical and Conceptual Narrative Review of Posture Assessment

**DOI:** 10.3390/jcm15187197

**Published:** 2026-09-16

**Authors:** Wojciech Michał Glinkowski, Robert Sitnik

**Affiliations:** 1 Center of Excellence “TeleOrto” for Telediagnostics and Treatment of Disorders and Injuries of the Locomotor System, Department of Medical Informatics and Telemedicine, Medical University of Warsaw, 02-091 Warsaw, Poland; 2 Polish Telemedicine and eHealth Society, 03-728 Warsaw, Poland; 3 Faculty of Mechatronics, Warsaw University of Technology, ul. św. Andrzeja Boboli 8, 02-525 Warsaw, Poland; robert.sitnik@pw.edu.pl

**Keywords:** body posture, postural development, anthropometry, auxology, posture classification, surface topography, rasterstereography, artificial intelligence, developmental trajectories, three-dimensional reconstruction, spinal sagittal alignment

## Abstract

**Background**: Posture assessment has progressed from visual observation and descriptive classification to quantitative measurement, three-dimensional imaging, and artificial intelligence (AI)-assisted analyses. Despite these advances, uncertainty persists regarding normative interpretation, developmental significance, and prognosis, particularly during growth and maturation phases. **Methods**: This historical and conceptual narrative review synthesizes literature from PubMed/MEDLINE, Scopus, Web of Science, Google Scholar, and Semantic Scholar, with additional citation tracking and targeted searches of historical monographs and foundational sources. The final literature search was completed on 30 May 2026. The review was narrative rather than systematic, and no formal risk-of-bias or evidence-quality appraisal was performed. Reporting and methodological transparency were strengthened by referencing the SANRA principles for narrative reviews. **Results**: The literature supports several overlapping methodological traditions: observation and classification, quantitative measurement, three-dimensional reconstruction, and automated image- and data-based analysis. These approaches have accumulated rather than replaced one another. Across these traditions, a persistent measurement–interpretation gap remains: postural configuration can be characterized by increasing precision, whereas developmental reference trajectories and prognostic interpretations remain limited. **Conclusions**: Developmental Postural Modeling (DPM) is proposed as a hypothesis-generating conceptual framework for future research, initially emphasizing posture during childhood and adolescence, when growth and maturation are most explicit. The DPM is not a validated clinical tool and does not currently provide individual predictions or treatment recommendations. Its proposed role is to organize future longitudinal research integrating morphology, function, development, environment, time, advanced imaging, and computational analysis.

## 1. Introduction

Human postural characteristics are influenced by growth, maturation, body proportions, and musculoskeletal development [1,2,3]. Postural characteristics reflect interactions among body proportions, musculoskeletal development, neuromuscular function, physical activity, and environmental influences [1,4]. Consequently, posture intersects with orthopedics, rehabilitation, physiotherapy, sports medicine, human biology, and public health [5]. Its clinical and societal importance has sustained scientific interest for more than a century [6,7,8]. In orthopedics and rehabilitation, postural assessment routinely evaluates spinal deformities, disorders, dysfunctions, and outcomes [9,10,11]. Within public health and school medicine, screening programs have been established to identify children at risk of postural abnormalities and spinal deformities [12,13,14]. Large-scale school screening programs have made postural and spinal assessment an established component of pediatric population screening [12,13,14]. Over time, assessment methods have been developed, from simple observation and classification systems to anthropometric measurements [7], photogrammetry [15], surface topography [16,17,18,19,20,21], raster stereography [22,23], and artificial intelligence-based approaches [24,25,26]. These developments have progressively expanded the capacity to quantify, document, and reproduce selected characteristics of postural configuration [10,11,27]. Notably, no universally accepted definition of a normal posture exists [4,5,28]. Numerous classification systems, measurement protocols, and imaging techniques have been proposed; however, disagreements persist regarding the normative criteria and clinically meaningful thresholds [10,11,27,29]. Evidence supporting the prognostic interpretation of many postural characteristics remains limited [10,30]. Simultaneously, most posture assessment methods rely on cross-sectional observations, although postural characteristics change during growth and maturation and therefore require developmental context for interpretation [10,29,31,32,33]. This highlights a paradox in postural research. A clinical posture assessment describes body configuration under defined conditions at a particular time point, whereas postural development refers to changes in postural characteristics across time, and postural control denotes the sensorimotor regulation of orientation and stability [3,32]. These constructs are related but not interchangeable. During childhood and adolescence, growth and maturation make the temporal dimension particularly explicit [1,2,32]. Thus, selected characteristics of postural configuration can be quantified with increasing technical precision, while the developmental significance of an individual observation often remains uncertain [11,34]. Historically, posture assessment has undergone several key conceptual and methodological transitions [1,2], progressively shifting from visual observation [6,7], and descriptive classification to quantitative measurement [10,22], three-dimensional reconstruction [23,35], and, more recently, artificial intelligence-assisted analysis [24]. Together, these epochs illustrate a major evolutionary trajectory from subjective descriptions to high-dimensional spatial reconstruction and automated, data-driven interpretation.

For the purposes of this review, posture denotes the observable configuration of body segments under specified assessment conditions, whereas postural alignment refers to the geometric relationships among those segments. Postural control is treated separately as the sensorimotor regulation of orientation and stability [3]. A developmental trajectory denotes a pattern of change in one or more postural characteristics across repeated observations. A developmental phenotype refers to an observed postural profile interpreted in relation to growth, maturation, function, environment, and time. The term digital phenotype refers to a phenotype characterized using digitally acquired longitudinal data, whereas spatial phenotype denotes a multidimensional geometric representation of body morphology. Developmental Postural Modeling (DPM) is the authors’ proposed hypothesis-generating framework for integrating these dimensions and is not a validated diagnostic, prognostic, or treatment-planning instrument.

Previous reviews have predominantly addressed specific components of posture assessment, including clinical measurement methods, photogrammetry, surface topography, three-dimensional imaging, scoliosis screening, and AI-assisted analysis [10,11,15,24,28,36]. Within the literature examined for this review, we did not identify a synthesis integrating the historical evolution of posture assessment with anthropological and auxological traditions, quantitative measurement, three-dimensional imaging, and emerging AI-assisted analysis within a developmental interpretative framework.

Across these methodological traditions, technological development has progressed more rapidly than frameworks for developmental interpretation. Quantitative measurement, surface reconstruction, three-dimensional imaging, and automated pattern analysis have substantially expanded the ability to characterize postural configuration, yet persistent questions concerning normality, developmental significance, and prognosis remain unresolved. At the same time, repeated digital assessment and increasingly multidimensional imaging provide new opportunities to characterize change in postural and morphological features across growth and maturation, while AI-assisted methods extend the capacity to analyze complex patterns within such datasets. Together, these developments expose a measurement–interpretation gap: increasingly detailed representations of postural configuration are available, but frameworks for interpreting their longitudinal developmental significance remain limited.

**Figure 1:**
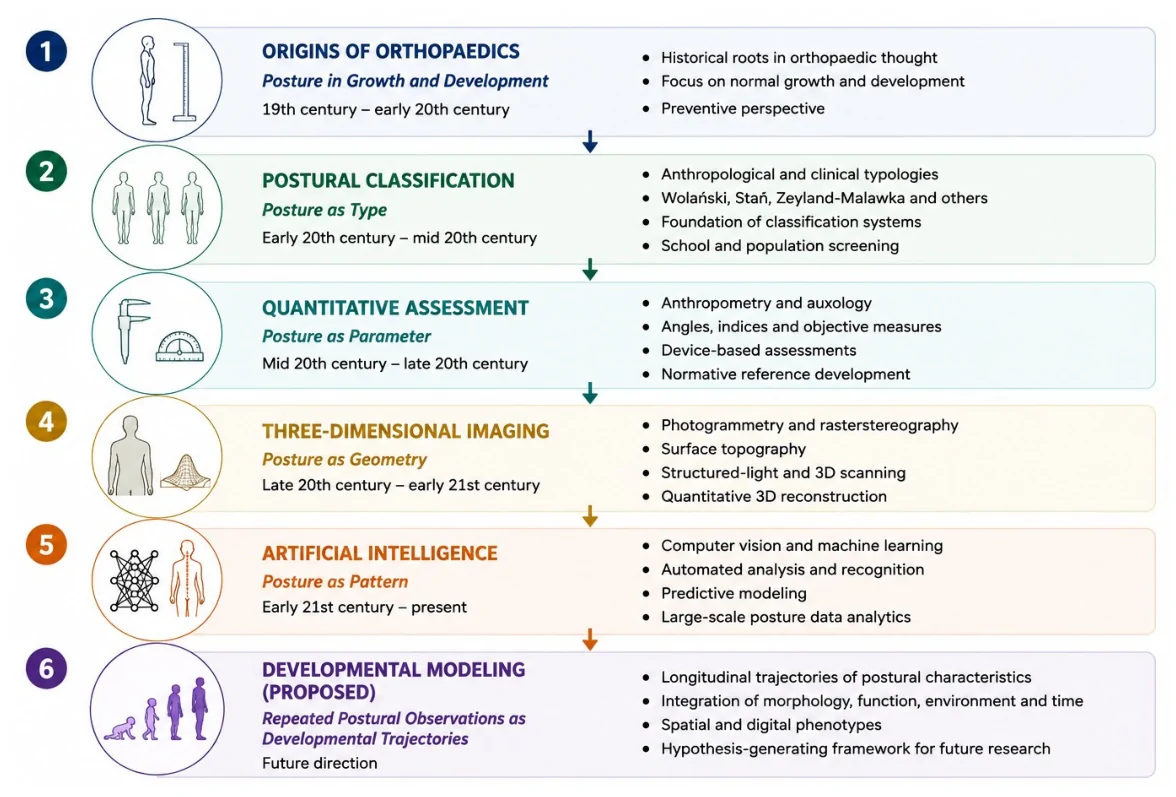
Conceptual Evolution of Posture Assessment: From Developmental Observation to Developmental Modeling. The figure summarizes six overlapping methodological and conceptual traditions: developmental observation (1), postural classification (2), quantitative assessment (3), three-dimensional imaging and reconstruction (4), AI-assisted pattern analysis (5), and the proposed longitudinal developmental interpretation based on repeated observations (6). These traditions accumulated and overlapped rather than sequentially replacing one another. The graphical rendering was produced with generative AI assistance based on the conceptual design and content specified by the authors.

Against this background, this historical and conceptual narrative review examines the major, overlapping traditions through which posture assessment has developed—from observation and classification to quantitative measurement, three-dimensional reconstruction, and automated analysis—and evaluates the persistent interpretative limitations associated with predominantly cross-sectional assessment paradigms (Figure 1). On the basis of this historical synthesis, we propose Developmental Postural Modeling (DPM) as a hypothesis-generating framework for future longitudinal research integrating biological variability, repeated postural observations, advanced imaging, and emerging computational approaches. Because the developmental evidence is strongest during periods of active growth and maturation, the proposed framework is focused primarily on children and adolescents; its extension to adulthood and aging remains a separate question requiring dedicated evidence.

## 2. Materials and Methods

### 2.1. Study Design

This study was a historical and conceptual narrative review. It was not designed as a systematic review of diagnostic accuracy, intervention effectiveness, or reliability of measurements. Its purpose was to synthesize major historical, conceptual, methodological, imaging, and computational traditions relevant to posture assessment and examine how these traditions inform the proposed DPM framework. The review was prepared with reference to the SANRA domains for narrative reviews, particularly the explicit justification of importance and aims, transparent description of literature searching, appropriate referencing, and clear separation of evidence from author interpretation [37].

This review was designed to analyze major conceptual transitions in the field, including the progression from developmental and anthropological interpretations of posture, through classification systems and quantitative measurements, to three-dimensional imaging technologies, automated analysis, and emerging developmental modeling frameworks. Particular attention was given to the distinction between posture assessed as a configuration at a defined time point, postural development as longitudinal change in postural characteristics, and postural control as the sensorimotor regulation of orientation and stability.

### 2.2. Literature Sources and Search Strategy

Literature searching was iterative and domain-based, consistent with the historical and conceptual purpose of this narrative review. PubMed/MEDLINE, Scopus, and Web of Science were used as the principal bibliographic databases. Google Scholar and Semantic Scholar were used as supplementary discovery sources, primarily for citation tracking, identification of historical and interdisciplinary literature, and retrieval of sources not readily identifiable through conventional database indexing. Searches were supplemented by backward and forward citation tracking, manual examination of reference lists, and targeted retrieval of historical monographs, original descriptions of methods and classification systems, consensus documents, methodological studies, and validation studies. The final literature search was completed on 30 May 2026, and publications available up to that date were considered. No publication-date restriction was otherwise applied.

The search strategy was organized around thematic domains rather than a single fixed query. Core concepts included posture assessment and postural development combined, as appropriate, with terms related to anthropometry, auxology, posture classification, sagittal alignment, photogrammetry, surface topography, raster stereography, structured-light imaging, three-dimensional reconstruction, artificial intelligence, machine learning, computer vision, pose estimation, longitudinal monitoring, trajectory analysis, and digital phenotyping. Supplementary Table S1 provides database-specific representative search strategies for the principal thematic domains. Because the original search process was iterative and not prospectively designed as a systematic-review search, these examples are provided to improve transparency and reproducibility and should not be interpreted as a retrospectively reconstructed, single, exhaustive search strategy.

The primary evidence base consisted of English-language literature. Selected Polish-language and other non-English sources were included when they provided historically or conceptually important information not adequately represented in the English-language literature. The authors assessed Polish-language sources in the original language. Other non-English sources were included only when their content could be evaluated through an accessible translation, an English-language abstract, or a reliable secondary scholarly source. Claims dependent on uncertain translation were not used as key evidence.

For historical and methodological source selection, publications described as influential, landmark, or historically important were identified using one or more of the following criteria: (1) original or early introduction of a concept, classification system, instrument, or imaging method; (2) demonstrable influence on subsequent methodological development or clinical adoption; (3) inclusion in consensus documents, guidelines, or major methodological reviews; (4) provision of important validation, reliability, normative, or developmental evidence; or (5) repeated citation across independent historical or methodological sources. Citation counts alone were not used as selection thresholds. Authors reached final judgments regarding conceptual relevance by consensus.

### 2.3. Eligibility Criteria

Publications were eligible if they contributed directly to one or more of the following domains:

- Historical and conceptual foundations of posture assessment;
- Anthropological, auxological, and developmental perspectives on posture;
- Posture classification systems and typologies;
- Clinical, anthropometric, photographic, photogrammetric, posturometric, or radiographic assessment methods;
- Three-dimensional non-invasive assessment technologies, including surface topography, raster stereography, structured-light imaging, and related approaches;
- Artificial intelligence, computer vision, smartphone-based systems, and automated posture-assessment systems;
- Methodological issues related to reliability, validity, reproducibility, standardization, and prognostic interpretation;
- Longitudinal, developmental, and population-based approaches to posture assessment and monitoring.

We considered original studies, reviews, methodological reports, historical analyses, consensus documents, and influential manuscripts. Publications were excluded when posture assessment was peripheral to the primary question; when they focused predominantly on balance, gait, postural control, occupational ergonomics, or neurological disease without a direct link to morphological posture assessment; when they addressed treatment outcomes without relevant assessment or monitoring information; or when they concerned adult spinal deformity or degenerative disease without informing the historical or methodological development of posture assessment. Studies on movement or postural control were retained only when they clarified the distinctions among posture, function, and sensorimotor control or informed the proposed conceptual framework.

Because the review focuses primarily on developmental interpretation during growth and maturation, the literature on adulthood and aging was not comprehensively synthesized. Therefore, claims regarding DPM are restricted to a proposed research framework, with pediatric and adolescent development as its principal initial application.

### 2.4. Narrative Synthesis

Publications were screened for their historical, conceptual, methodological, imaging, developmental, and clinical relevance to the aims of the review. Formal risk-of-bias assessment and quantitative evidence synthesis were not performed because the review was designed as a historical and conceptual narrative review rather than a systematic review. Both authors contributed to the identification and evaluation of key sources and to the interpretation of the historical, methodological, and technological literature. Differences in judgments regarding conceptual relevance or inclusion were resolved through discussion and consensus.

The literature-identification process was iterative and domain-based and incorporated database searching, backward and forward citation tracking, examination of reference lists, historical monographs, and targeted retrieval of foundational and methodological sources. Because the review was not prospectively designed as a systematic review, a complete deduplicated record-level search log and formal screening flow were not maintained from the outset. Consequently, exact retrospective counts of records identified, deduplicated, screened, excluded, and retained cannot be reported reliably and were not reconstructed post hoc.

To improve methodological transparency, the databases and supplementary discovery sources, final search date (30 May 2026), principal search domains, eligibility and exclusion criteria, source-selection principles, and representative database-specific search strategies are reported.

The synthesis was structured around eight thematic domains.

- Anthropological and developmental foundations;
- Posture classification systems;
- Quantitative measurement approach;
- Photographic and photogrammetric methods;
- Surface topography and three-dimensional reconstruction;
- Artificial intelligence and automated assessments;
- Unresolved methodological and interpretative challenges;
- Developmental postural modeling, and future directions.

This review focused on recurring challenges identified in the literature, including reliance on single-time-point assessments, single-plane measurements, single-parameter interpretations, heterogeneous definitions of normal posture, limited longitudinal evidence, and the absence of a developmental reference framework.

To facilitate the interpretation of the historical evolution described throughout this review, the major stages of posture assessment were organized into a chronological framework summarizing the dominant paradigms, primary objects of analysis, methodological approaches, and principal research questions associated with each era (Table 1).

The final stage (Developmental Modeling Era) is presented as a conceptual framework by the authors based on the historical synthesis in this review. This should not be interpreted as an established classification of the postural assessment paradigms.

The following sections examine each stage in greater detail, highlighting both the methodological advances and the evolving conceptual understanding of posture.

### 2.5. Development of the Conceptual Framework

Based on the narrative synthesis, a conceptual framework was developed to describe the transition from predominantly cross-sectional posture assessment toward developmental postural modeling. The framework is not intended as a validated diagnostic model but rather as a structured research perspective that integrates the principal themes identified in the literature review.

The framework incorporates five interrelated dimensions.

- Morphology: Body alignment, spinal curvature, trunk asymmetry, and three-dimensional geometry.
- Function: Postural control, movement adaptation, biomechanical performance, and sensorimotor integration.
- Development: Growth, maturation, biological age, and developmental variability.
- Environment: Physical activity, lifestyle, behavioral influences, and contextual factors.
- Time: Longitudinal monitoring, developmental trajectories, and temporal dynamics of postural change.

This framework was subsequently used to explore how posture assessment may extend from single-time-point description and classification toward longitudinal developmental interpretation.

Generative artificial intelligence (ChatGPT, OpenAI (GPT-5.5; OpenAI, San Francisco, CA, USA)) was used during manuscript preparation to assist with language and structural editing and with the graphical implementation of Figures. The authors defined the scientific concepts, content, structure, and interpretation represented in these figures. The authors reviewed, revised, and approved AI-generated outputs, and did not use them for literature selection, data extraction, evidence appraisal, statistical analysis, or autonomous scientific interpretation.

## 3. Anthropological Foundations

### 3.1. Origins of Orthopaedics and Developmental Observation

The origins of posture assessment can be traced back to the foundation of orthopedics. The French physician Nicolas Andry introduced the term Orthopaedia in 1741 [6]. Andry explicitly derived the neologism from Greek roots denoting “straight” (orthos) and “child” (pais/paidos), as discussed in later historical scholarship [66]. Therefore, we avoid treating “Orthos Paidos” as a grammatically independent Greek expression. Orthopedics was originally conceived not as a surgical discipline but as a field dedicated to preventing and correcting deformities during growth and development. Within this historical framework, body posture was regarded as a visible manifestation of musculoskeletal development and an important indicator of a child’s overall physical condition [1,2,39,40,67]. Consequently, early orthopedic concepts were closely linked to prevention, education, and developmental observations [1,6,39,67]. The primary objective was not merely to address established deformities but to identify deviations during growth and implement preventive or corrective measures [68]. This developmental perspective has shaped posture assessment for more than two centuries and remains relevant today, despite technological advances that have transformed diagnostic capability. Importantly, the early orthopaedic paradigm emphasized growth, prevention, and developmental observation rather than quantitative anatomical measurement [6,66]. Postural characteristics change throughout childhood and adolescence and are influenced by biological maturation, environmental conditions, physical activity, and functional adaptation [1,2,39,40]. By the end of the nineteenth century, the study of posture was increasingly extending beyond its original orthopedic context. It was becoming associated with broader interests in human growth, physical development, and body function [1,39,40].

### 3.2. Posture as a Developmental Characteristic

Long before the emergence of modern imaging technologies, posture was predominantly regarded as a developmental characteristic reflecting the interaction between growth, maturation, body proportions, neuromuscular control, and environmental influences [1,39,67,69]. Within anthropological and auxological traditions, postural characteristics were interpreted in relation to growth and maturation rather than regarded as fixed anatomical attributes independent of developmental stage. Growth-related changes in body proportion substantially influence postural alignment [1,3,67].

Variations in trunk length, lower limb proportions, pelvic morphology, muscle development, and maturation of postural control mechanisms contribute to age-dependent modifications of spinal curvatures and overall body configuration. Consequently, a postural configuration observed at a particular age represents a time-specific observation that can be interpreted developmentally only in relation to age, maturation, and—where available—repeated longitudinal observations.

Although cross-sectional examinations provide valuable information regarding current body alignment, they offer limited insight into developmental variability and future adaptation. Two children presenting with similar postural characteristics at a given age may subsequently follow markedly different developmental pathways owing to differences in growth velocity, maturation timing, physical activity, body composition, and neuromuscular function [1,3,32,39,67]. Anthropological research has demonstrated substantial interindividual variability in growth and somatic development. From this perspective, the question is whether posture and observed characteristics are appropriate for the individual’s developmental stage. This distinction matters because many postural features in childhood may be transient developmental phenomena rather than permanent abnormalities [3,30,32]. Fluctuations in spinal curvature, trunk symmetry, shoulder alignment, and pelvic position during growth indicate developmental context matters for interpreting postural findings. This developmental perspective founded anthropological and preventive orthopedic approaches. Rather than seeking an ideal configuration for all individuals, these traditions emphasize developmental diversity, biological variability, and age-specific patterns. These concepts influenced classification systems and inform individualized assessments, normative values, and longitudinal monitoring [7,39].

Despite advances in imaging and three-dimensional reconstruction, interpreting posture in its developmental context remains unresolved. Observed postural configuration should therefore be interpreted within its developmental context rather than solely as a geometric arrangement of body segments.

### 3.3. Anthropological and Auxological Perspectives

The scientific study of posture has gradually expanded beyond the original orthopedic focus on deformity prevention and has become increasingly integrated with anthropology, human biology, and auxology. These disciplines emphasize that growth and development involve substantial biological variability and that morphological characteristics should be interpreted within the context of normal developmental diversity rather than solely through the lens of pathology [1,2,39,40,67].

Auxology, the science of human growth and development, introduced a population-based perspective for evaluating characteristics. Rather than viewing posture as normality or deformity, auxological approaches examine the distribution, variability, and determinants of developmental traits within populations. This shift has implications for posture assessment, encouraging interpretation of characteristics in the context of growth and maturation [1,67,69].

Twentieth-century anthropological research has shown that body proportions, growth, maturation, nutrition, physical activity, and socio-environmental conditions substantially contribute to variability in musculoskeletal development. Consequently, postural characteristics in children and adolescents cannot be understood without reference to the developmental processes shaping them. These observations challenge the notion that a single postural configuration can serve as a universal reference standard [1,3,67].

Particularly influential in this regard were the contributions of researchers such as Ćwirko-Godycki [69], Przewęda [70], and Wolański [2,39,40] and their collaborators, who emphasized the importance of biological age, growth dynamics, longitudinal development, and population variability in interpreting human morphology. Within this conceptual framework, observed postural characteristics were increasingly interpreted as manifestations of interactions among skeletal growth, muscular adaptation, neuromotor control, and environmental influences.

This perspective differs from later measurement-oriented approaches focused on isolated anatomical parameters. Anthropological investigations described the current state of body alignment and sought to understand developmental mechanisms producing observed postural patterns [1,39,40,67]. As a result, postural configurations were interpreted in relation to developmental stage and biological maturation rather than a fixed geometric standard.

A consequence of this approach was recognizing biological variability as inherent to human development rather than a deviation from an idealized norm [1,39,40,67,71]. Consequently, developmental diversity has become scientifically interesting. The concept that multiple postural patterns may coexist in healthy populations remains relevant today, as posture assessment struggles with the absence of universally accepted normative definitions and clinically meaningful reference standards [4,30,71]. Therefore, anthropological and auxological traditions introduced concepts resonating in postural research, including developmental trajectories, biological variability, population diversity, and distinctions between biological and chronological age. These ideas influenced classification systems and discussions of individualized assessment and developmental modeling [39,40,70,71,72].

## 4. The Era of Postural Classification Systems

The growing recognition of biological variability in human posture has created a need for frameworks that can systematically describe recurring postural patterns. Posture classification systems emerged as one of the earliest responses to this challenge and represent an important conceptual transition from descriptive observation to a structured interpretation of postural diversity.

### 4.1. Why Classify Posture?

The recognition of considerable diversity in human posture has created fundamental challenges for clinicians, anthropologists, educators, and rehabilitation specialists [1,39,40,67,69]. If posture varies because of growth, maturation, body proportions, and environmental influences, a framework is needed to describe this diversity systematically and clinically.

The emergence of posture classification systems represents one of the earliest attempts to address this issue [7,70]. Rather than focusing on anatomical measurements, these systems identified recurring postural patterns that could be recognized, categorized, and compared across individuals and populations. Classification is therefore an important step between descriptive observations and measurement-based approaches. Historically, classification systems facilitated communication among practitioners, supported school-based screening, guided preventive interventions, and described population-level patterns of postural development in children [13,14,73]. More importantly, they reflected recognition that a simple dichotomy of normal and pathological posture could not adequately describe posture. By organizing observed postural diversity into recognizable categories, classification systems provided a practical framework for interpreting biological variability before objective measurement technology became widespread. Among the earliest influential efforts were classification systems developed in early twentieth-century Europe, particularly the work of Staffel [7], whose typological framework laid the foundation for describing recurrent postural patterns and later influenced both clinical and educational approaches to posture assessment.

### 4.2. Early European Classification Approaches

The first systematic attempts to classify posture emerged in Europe during the late nineteenth and early twentieth centuries [5,7]. These approaches relied on visual observation and focused on the body’s sagittal profile. Investigators sought recurring configurations of spinal curvatures, trunk alignment, and body proportions for systematic description and comparison across individuals and populations.

Among the most influential early systems was the classification proposed by Staffel [7], which distinguished several characteristic postural types according to the configuration of thoracic kyphosis (TK) and lumbar lordosis (LL). Rather than treating posture as a binary state of normality or deformity, Staffel’s approach recognized it as a continuum of recurring configurations organized into a coherent typological framework. This study introduced the idea that postural variability could be grouped into recognizable categories, providing a practical framework for understanding population-level differences in body alignment. Similar systems emerged in European countries, reflecting growing interest in preventive orthopedics, developmental medicine, and population-based approaches to physical development. Despite subjective assessments and nonstandardized criteria, these classifications were an important conceptual advance, framing posture as a spectrum of recognizable patterns and transforming postural diversity into interpretable categories for clinical, educational, and population studies [5]. Although largely qualitative, these early approaches laid the foundation for later quantitative methods by showing that postural diversity could be organized into recurring, clinically interpretable patterns, prompting simplified classification systems for large-scale screening and population-based research.

### 4.3. The Polish Developmental Classification Tradition

A particularly distinctive contribution to posture assessment emerged from Polish anthropological, orthopedic, and physical-education traditions during the second half of the twentieth century. This Polish auxological and orthopedic school developed in parallel with Western European and American classification systems, placing unique emphasis on physiological individual variability in healthy children rather than focusing exclusively on pathology. Unlike many contemporary classification systems, which primarily focus on identifying postural abnormalities and deformities, Polish investigators have developed frameworks to describe the diversity of postural patterns observed in healthy children and adolescents.

This developmental perspective was strongly influenced by preventive orthopedics, human biology, and auxology. Among the most influential contributors were Wiktor Dega [38], Napoleon Wolański [2,39], Ewa Zeyland-Malawka [41], and Tadeusz Kasperczyk [74], as well as other representatives of the Polish developmental traditions.

Collectively, their work established a distinctive developmental tradition of posture assessment at the intersection of orthopedics, anthropology, human biology, and physical education [38,39,40,41,70]. Wolański’s typological system, which categorizes posture into kyphotic, balanced, and lordotic types based on the interaction between the thoracic and lumbar curves, has been particularly influential [39,40]. Rather than defining posture using a single normative standard, this approach sought to describe the diversity of postural patterns observed throughout an individual’s growth and development. Subsequent modifications proposed by Zeyland-Malawka [41] were based on the simplification and refinement of Wolański’s classification system [39,40], making it more applicable to population studies and practical postural assessments. While preserving the developmental foundations of the original framework, the revised approach introduced more accessible and practical evaluation criteria than the original. Furthermore, it emphasizes biological variability, age-dependent changes, and the existence of multiple physiologically acceptable postural configurations in healthy subjects. Importantly, this tradition challenges the concept of a universally applicable model of “correct posture.” Zeyland-Malawka explicitly argued that no single postural standard could adequately represent all ages, developmental stages, or populations and that posture should be interpreted within its biological and developmental context. Consequently, Polish developmental classification systems interpreted postural configurations in relation to growth and maturation rather than against a single fixed anatomical standard [39,40,70]. Supplementary Table S2 presents a broader comparison of major posture assessment concepts and methods, along with their historical contributions. This perspective anticipated several concepts that have re-emerged in contemporary postural research, including developmental variability, individualized assessment, longitudinal monitoring, and within-age and development-dependent patterns, rather than relying solely on cross-sectional observations.

### 4.4. Strengths of Classification-Based Approaches

Classification systems provide a practical way to organize postural diversity into recognizable categories without specialized equipment, supporting communication, school screening, epidemiological studies, and population comparisons [12,14,39,40,41]. Conceptually, they represent an early form of structured pattern recognition: rather than reducing posture to a normal/pathological dichotomy, they acknowledge recurring configurations and biological variability. This contribution established a foundation for subsequent quantitative and technology-assisted assessments.

### 4.5. Limitations of Classification-Based Approaches

Their principal limitations are subjectivity, limited reproducibility, and inability to quantify subtle differences. Even developmentally informed classifications are usually applied at a single time point and therefore provide limited information about temporal change or individual trajectories.

These limitations stimulated a transition from descriptive classification to objective measurement. Supplementary Table S3 summarizes the evolution of the principal scientific and clinical questions addressed by the successive approaches.

## 5. The Quantification Era

### 5.1. From Description to Measurement

The limitations of visual classification systems have gradually stimulated the search for more objective methods of postural assessment [7,8,39,40,41]. As clinical, educational, and research applications expanded, emphasis on improving reproducibility, precision, and comparability of postural evaluations increased. The challenge shifted from recognizing recurring postural patterns to objectively measuring and quantifying them clinically. This transition reflects developments in medicine and biology, where standardized measurements supplement health observations [1]. In posture assessment, investigators have sought ways to convert qualitative descriptions into numerical variables comparable across individuals, populations, and time points. This shift to quantitative assessment moves beyond predefined postural categories, emphasizing measurement of anatomical and geometric characteristics, including spinal curvatures, trunk proportions, body segment alignment, and symmetry [10,27,32,42,43,44,75,76]. This approach enabled more precise documentation of postural characteristics and provided a foundation for statistical analyses and population-based research.

Importantly, quantification does not replace classification; rather, it complements it [39,40,41]. Many quantitative methods rely on concepts developed within classification systems while introducing objective measures and reducing observer dependency. Thus, the quantitative era represents an extension of earlier efforts to organize and interpret postural variability.

### 5.2. Anthropometric and Clinical Measurements

Early efforts to quantify posture relied primarily on anthropometric and clinical measurements. Building on the conceptual frameworks established by posture classification systems, investigators have sought objective methods to evaluate body proportions, spinal curvatures, trunk symmetry, and segmental alignment. These approaches reflect a growing desire to reduce observer dependency and improve the reproducibility of postural assessments.

Anthropometric methods utilize linear, angular, and proportional measurements obtained from the anatomical landmarks [1,39,40]. Parameters such as trunk length, shoulder and pelvic alignment, limb proportions, and spinal curvature characteristics have been increasingly incorporated into postural evaluation protocols. Standardized measurement procedures have facilitated comparisons between individuals, age groups, and populations.

Clinical assessment methods have evolved in parallel with these advances. Visual inspection was supplemented by goniometric measurements, plumb line evaluation, inclinometry, and other techniques designed to provide quantitative descriptions of postural characteristics [10,28,29,43,77,78]. These methods enable clinicians to document deviations more precisely and to monitor changes over time.

A major advantage of quantitative assessment is its ability to generate numerical data suitable for statistical analysis. This development has significantly expanded opportunities for epidemiological studies, normative research, and investigations into developmental variability. Quantification also facilitates the establishment of reference values and population-based comparisons, thereby strengthening the scientific foundation of posture assessment [1,39,40,70].

However, despite improvements in objectivity and reproducibility, anthropometric and clinical measurements remain subject to significant limitations. Many methods depend on external anatomical landmarks, are susceptible to measurement errors, and provide only simplified representations of the inherently three-dimensional body geometries. Most importantly, quantitative measurements describe postural characteristics with increasing precision but provide limited insight into the developmental processes underlying the observed patterns.

### 5.3. Postural Indices and Quantitative Models

As posture assessment has become increasingly quantitative, researchers have sought methods to summarize complex anatomical relationships into standardized numerical parameters [1,39,40,70]. This led to the development of numerous postural indices, angular measurements, proportional ratios, and composite scoring systems intended to describe body alignment reproducibly and comparably.

The rationale behind these approaches is simple. If posture can be expressed numerically, individuals, populations, and developmental stages can be compared objectively. Therefore, quantitative indices are expected to facilitate epidemiological investigations, normative studies, clinical monitoring, and evaluation of intervention results [1,39,40,70]. Various systems have been proposed to characterize spinal curvatures, trunk proportions, body symmetry, sagittal alignment, and overall posture quality [39,40,42,44,74].

The introduction of quantitative models is a methodological advance. Numerical parameters enable statistical analysis, population-based comparisons, and reference ranges not achievable with purely descriptive classification systems, transforming posture assessment from an observational discipline into a measurable domain. However, their increasing availability has created challenges, as investigators often use different anatomical landmarks, measurement protocols, and reference standards, causing heterogeneity among studies [10,75]. Consequently, posture assessment has become more precise but often less conceptually integrated.

Most quantitative models have remained focused on describing postural characteristics at a single time point rather than their developmental change. Numerical measurements indicate curvature, asymmetry, or deviation; however, they provide limited information about the developmental mechanisms underlying these observations or their future trajectories. Thus, although quantification improves objectivity, it does not resolve the challenge of interpreting postural characteristics within the context of growth and maturation.

In retrospect, many quantitative approaches were precursors to modern computational analyses, creating conditions for digital assessment methods, three-dimensional reconstruction techniques, and artificial intelligence-based analytical tools [10,42,44,75].

### 5.4. Limitations of the Quantitative Paradigm

Quantification improves objectivity but introduces new problems. Measurements depend on the selected landmarks, protocols, and reference standards, limiting comparability [10,75,79]. Moreover, posture was frequently reduced to isolated angles, distances, or indices, whereas many methods remained predominantly two-dimensional [10,42,44].

Greater numerical precision does not resolve developmental interpretation. Single-time-point measurements describe configuration but not variability, adaptation, or trajectories. These limitations have encouraged photographic, photogrammetric, and three-dimensional approaches for more comprehensive posture representation.

## 6. The Imaging and Reconstruction Era

### 6.1. From Measurement to Visualization

The limitations of conventional anthropometric and clinical measurements have spurred the development of methods that can capture posture more comprehensively [10,75]. While quantitative assessment provides numerical descriptions of anatomical characteristics, it is limited in representing the human body’s complex three-dimensional organization. As posture research has advanced, attention has shifted toward techniques that visualize and reconstruct body geometry rather than quantify isolated parameters. This reflects a shift in posture assessment: the objective extends beyond measuring distances, angles, or proportions to understanding spatial relationships among anatomical structures, with researchers seeking methods that represent body alignment as an integrated geometric configuration rather than a set of measurements.

The emergence of photographic documentation, surface visualization techniques, and later three-dimensional reconstruction methods has fundamentally transformed postural assessment [8,16,22,63]. For the first time, investigators can analyze body shape, trunk symmetry, spinal contours, and spatial alignment in ways difficult or impossible with conventional methods. These developments expanded information for postural evaluation and created opportunities for documentation and monitoring. Importantly, the transition from measurement to visualization began postural assessment as an image-based discipline. Rather than relying on numerical parameters, investigators increasingly used visual and geometric representations of body morphology, establishing the foundation for image analysis, automated pattern recognition, and artificial intelligence-assisted postural assessment [63,64,80]. Supplementary Table S2 provides a comparative overview of the principal technologies, measurement principles, outputs, dimensionality, and conceptual contributions discussed in this review.

### 6.2. Early Imaging Approaches

The introduction of photography represented one of the first major advances in image-based postural assessments [8]. Unlike traditional visual inspection, photographic methods enable permanent documentation, repeated analyses, and comparisons over time. Standardized photographic protocols have been gradually incorporated into clinical, educational, and research settings, facilitating a more systematic evaluation of body alignment and postural characteristics [63].

Early imaging approaches primarily focused on two-dimensional representations of body alignment and external morphology [15,63,81,82,83]. The investigators analyzed sagittal and frontal body profiles, examined anatomical landmarks, and assessed the alignment using reference lines, grids, and proportional relationships. These techniques improve documentation and reduce reliance on memory-based clinical observations, thereby enhancing reproducibility and facilitating longitudinal comparisons.

Despite these advantages, conventional photographic methods remain limited by projection-related distortions and their inability to fully capture three-dimensional body geometry. Consequently, researchers have increasingly sought methods that can reconstruct surface morphology and spatial relationships more accurately, leading to the development of photogrammetry, surface topography, and other non-invasive imaging technologies.

### 6.3. Photogrammetry and Surface Topography

The limitations of conventional photographic assessments have stimulated the development of techniques that extract quantitative information from visual representations of the body [10,75,79,84]. Among the most influential advances were photogrammetry and surface topography, which introduced new possibilities for the objective analysis of posture based on external body morphology [16,22,49,63].

Photogrammetric methods enable investigators to derive spatial measurements from photographic images by identifying the anatomical landmarks and geometric relationships. [10,63,75]. Compared with traditional clinical measurements, photogrammetry offers improved reproducibility, permanent image documentation, and the ability to analyze multiple postural parameters simultaneously. These advantages have contributed to its widespread application in posture research, epidemiological studies, rehabilitation, and musculoskeletal assessment.

Surface topography further advanced the field, including techniques such as Moiré topography and rasterstereography, which enabled increasingly sophisticated characterizations of trunk morphology and surface geometry [16,22]. Rather than focusing on individual measurements, surface topographic methods characterize trunk and body geometry. By analyzing contours, asymmetries, and spatial relationships across anatomical regions, they provide a more comprehensive representation of posture than conventional anthropometry or two-dimensional photography.

Importantly, photogrammetry and surface topography shifted posture assessment from parameter-based measurements to geometric reconstruction. Analysis no longer centered on isolated angles or distances but on the spatial organization of body structures and their relationships. This marked a step toward representing postural configuration as a complex geometric pattern rather than a set of independent variables.

These developments also expanded image-based analysis in posture research. The availability of digital images and geometric representations created opportunities for computerized processing, automated parameter extraction, and sophisticated pattern-recognition approaches. Many concepts underlying computer vision and artificial intelligence-assisted posture assessment can be traced to foundations established by photogrammetry and surface topography.

A conceptual transition occurred when posture assessment moved from two-dimensional representations referenced to gravity-based vertical alignment and planar projections to three-dimensional reconstruction within Cartesian spatial coordinate systems (Supplementary Table S2). Researchers increasingly characterized postural configuration as a spatial geometric structure defined by relationships among points, surfaces, and volumes rather than body segments relative to a plumb line or single observation plane.

Subsequent technological developments differed mainly in how body geometry was acquired and reconstructed, progressing from two-dimensional image analysis to surface mapping, three-dimensional reconstruction, and digital representations of human morphology.

Nevertheless, although these methods improve visualization and quantification of external body morphology, developmental questions remain largely unanswered.

### 6.4. Raster Stereography and Three-Dimensional Reconstruction

The development of moiré topography, raster stereography, structured-light systems, and related three-dimensional surface imaging techniques represent successive milestones in the evolution of posture assessment [16,18,22,49,53,57,58,62,85,86,87]. These technologies have expanded ability to capture and reconstruct body morphology while avoiding exposure to ionizing radiation, making them attractive for longitudinal monitoring and pediatric applications.

Unlike conventional photogrammetry, which relies on measurements from two-dimensional images, three-dimensional surface imaging enables reconstruction of trunk geometry and body configuration. By projecting optical patterns onto body surface and analyzing deformation, these systems generate representations of posture, including trunk asymmetry, spinal contours, shoulder and pelvic alignments, and body shape [22,49,57].

A major advantage of three-dimensional reconstruction is its capacity to capture postural configuration as a spatial geometric phenomenon [22,23,88]. Rather than describing isolated anatomical parameters, investigators can analyze relationships among multiple regions, providing a comprehensive representation of postural organization. This shift reflects a holistic approach to posture assessment, emphasizing geometry, morphology, and pattern analysis.

These technologies digitized posture assessment. Surface reconstructions can be stored, processed, and compared, enabling documentation and longitudinal observation of postural changes. The datasets exceeded anthropometric measurements and opened possibilities for computational analysis.

Three-dimensional reconstruction marked the transition from parameter-based to geometry-based assessment. Unlike earlier approaches referenced to plumb lines, planar projections, or anatomical landmarks, it represented posture in a Cartesian coordinate system. The object of analysis is no longer an angle, index, or distance but a representation of body morphology. This transition provided a methodological foundation for subsequent computer-vision and multidimensional computational analyses, in which body characteristics can be represented as complex geometric datasets.

Nevertheless, despite body-geometry representations, three-dimensional imaging has not resolved developmental interpretation. Surface reconstructions characterize geometry in detail but do not explain why a configuration emerged, how characteristics change over time, or an individual’s developmental trajectory [23,54,56]. Consequently, the growing sophistication of reconstruction technologies further highlights the distinction between accurate descriptions and meaningful interpretation.

### 6.5. Digitalization, Pattern Recognition, and Computational Analysis

The widespread adoption of digital imaging technologies has fundamentally transformed posture assessment. As photographic, photogrammetric, and three-dimensional reconstruction systems have become increasingly computerized, postural data are no longer limited to visual observations or isolated measurements. Instead, body morphology can be represented as a structured digital dataset containing numerous geometric and spatial variables.

This transition has created new opportunities for computational analyses. For the first time, posture can be represented not only as geometry, but also as data amenable to automated processing and large-scale statistical analysis. Digital posture representations enabled the automated extraction of anatomical landmarks, calculation of geometric parameters, assessment of symmetry, and comparison of complex morphological patterns. As computational capabilities have expanded, posture assessment has increasingly shifted from manual evaluation to algorithm-assisted analysis [49,54,55,56].

A significant development was the emergence of pattern-based approaches. Rather than focusing on individual measurements, investigators have begun analyzing relationships among multiple anatomical features simultaneously. This allowed postural configurations to be represented as multidimensional patterns characterized by geometric interactions rather than independent variables.

The growing availability of digital postural datasets has facilitated longitudinal analyses and large-scale population studies. Researchers can compare thousands of observations, identify recurring postural patterns and emerging postural phenotypes, and explore variability across developmental stages, populations, and clinical conditions [11,12,13,14,39,40,45,71,76]. These developments have significantly expanded the analytical possibilities in postural research.

Many concepts later central to machine learning and artificial intelligence-assisted image analysis emerged during this period. Automated feature extraction, pattern recognition, multidimensional classification, and computational modeling have been integrated into posture assessment, establishing methodological foundations for AI-based approaches [59,63].

Despite increasing computational sophistication, most analytical frameworks still focus on descriptions, classifications, and detections. Although digital technologies better identify postural patterns, understanding their developmental meaning, biological significance, and future trajectories remains unresolved.

## 7. Artificial Intelligence and Automated Pattern Recognition

### 7.1. From Human Observation to Automated Pattern Recognition

Many concepts that underpin contemporary AI-assisted posture assessment—including pattern recognition, phenotype identification, population variability, and classification of postural characteristics—have historical roots in earlier anthropological, developmental, and classification traditions [1,39,40,41]. However, digital datasets, advances in medical imaging, and increasing computational power have expanded the scale of in vivo investigation of these concepts.

Therefore, the history of posture assessment can be viewed as a progression from observation and pattern recognition to increasingly automated image-based analysis [36,89]. Early observers identified postural configurations by inspection. Later, classification systems organized patterns into typologies, and quantitative methods introduced objective measurements of characteristics. Subsequent developments in photogrammetry, surface topography, and three-dimensional reconstruction transformed representation of postural configuration into increasingly digital, geometrically quantifiable forms [36,56,57,90]. Artificial intelligence is the latest stage in this development, enabling automatic recognition and analysis of complex posture patterns. AI-assisted posture assessment can be considered a return to pattern recognition, although performed through computation rather than by human observers [24,91].

Unlike traditional methods, which rely on predetermined measurements or expert-defined classification criteria, machine learning algorithms can identify relationships in datasets and detect patterns not immediately apparent to human observers, including multidimensional geometric representations that exceed practical limits of human visual interpretation [24,91].

This capability has stimulated growing interest in AI-assisted posture assessment in orthopedics, rehabilitation, sports medicine, and movement science [24,91,92].

AI-based systems can extend automated assessment by identifying complex relationships among anatomical, geometric, functional, and imaging-derived variables. Thus, artificial intelligence may offer new opportunities to analyze and interpret postural variability beyond measurement-based approaches [24,91].

### 7.2. AI Applications in Posture Assessment

Contemporary applications of artificial intelligence in posture assessment encompass a broad range of image- and sensor-based tasks, including automated anatomical landmark detection, body segmentation, posture classification, asymmetry assessment, scoliosis screening, movement analysis, and musculoskeletal abnormality prediction. These applications use diverse data sources, such as photographic images, video recordings, depth sensor data, three-dimensional surface reconstructions, and other forms of digital body imaging [24,91].

Machine learning and deep learning techniques have increasingly been applied to the automated identification of postural features and abnormalities [93,94]. Convolutional neural networks, computer vision systems, and markerless pose estimation frameworks have been used to detect spinal deformities, assess body alignment, estimate postural parameters, classify postural patterns, and analyze movement-related features of the human body [93,94,95,96]. Compared with traditional assessment methods based on predefined measurements, AI-based systems can simultaneously analyze multiple geometric, spatial, and image-derived features, enabling the exploration of increasingly complex multidimensional relationships within posture datasets [26,95,96].

A particularly important development is integrating artificial intelligence with image-based posture assessment. Digital imaging technologies provide structured visual and geometric data, whereas automated analytical algorithms enable feature extraction, pattern recognition, classification, and predictive modeling. This combination may improve efficiency, reduce observer dependency, and support large-scale screening and monitoring programs [96]. More broadly, posture assessment is increasingly incorporating computer-assisted image analysis, computational morphology, and multidimensional data analysis.

### 7.3. Opportunities and Current Limitations

Most contemporary AI applications remain focused on detection, classification, segmentation, and measurement [93,94,95,96]. Accurate pattern recognition does not by itself establish biological meaning, developmental origin, future evolution, or clinical relevance [1,39,40,41].

AI expands capacity but does not eliminate the measurement–interpretation gap. Integration of developmental biology, longitudinal observation, growth dynamics, and predictive modeling remains limited [25,80,97].

### 7.4. Beyond Recognition: Toward Developmental Modeling

From a historical perspective, artificial intelligence may be viewed as a continuation of the long-standing effort to improve the detection and characterization of postural patterns. However, the increasing availability of longitudinal datasets, population-scale observations, and advanced computational techniques creates opportunities that extend beyond pattern recognition alone [1,24,31,38,39,40,96,98].

Future developments may enable postural configurations observed at individual time points to be analyzed as repeated observations within developmental trajectories across growth and maturation [39,40,41]. This approach requires integrating imaging data, longitudinal observations, biological variability, developmental trajectories, and predictive analytics into a unified conceptual framework [1,39,40].

In this context, artificial intelligence should not be viewed as the final stage in the evolution of postural assessments. Rather, it may serve as a technological foundation for more comprehensive developmental modeling frameworks. Therefore, the transition from automated pattern recognition to developmental interpretation may be the next major challenge in posture research.

**Figure 2:**
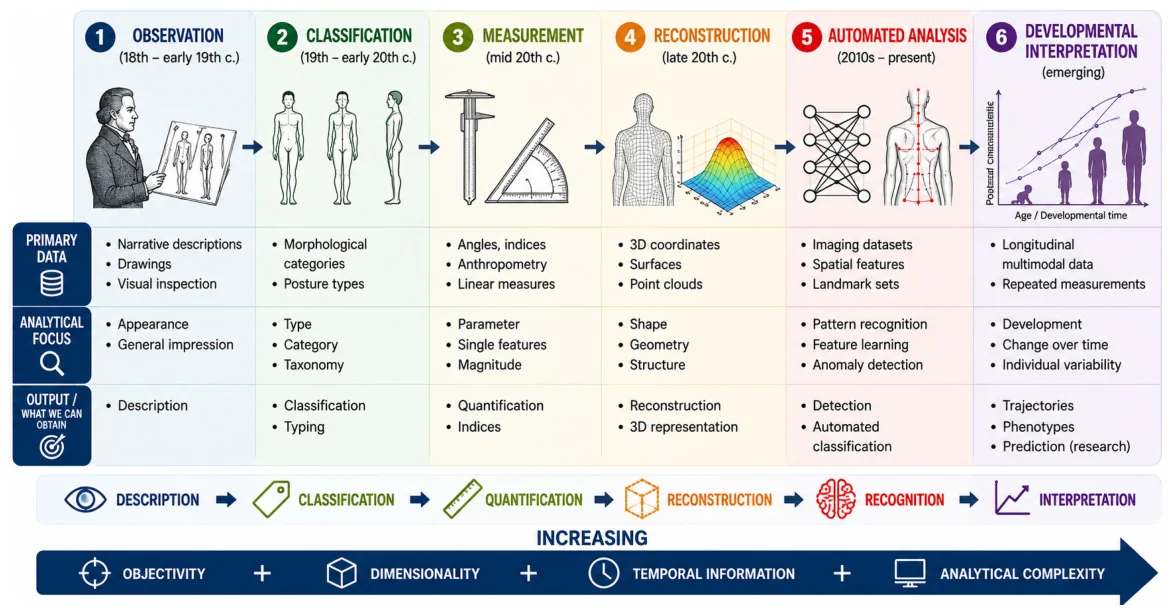
Evolution of Postural Data and Analytical Complexity: From Qualitative Observation to Longitudinal Developmental Interpretation. The graphical rendering was produced with generative AI assistance based on the conceptual design and content specified by the authors.

Across successive methodological eras, posture assessment has evolved not only through increasing measurement precision but also through the progressive expansion of data complexity, dimensionality, and analytical capabilities. This transition transformed posture assessment from qualitative descriptions to increasingly sophisticated forms of quantitative, spatial, and computational analyses (Figure 2).

## 8. The Unresolved Challenge: Interpretation Versus Measurement

### 8.1. The Measurement–Interpretation Gap

Throughout its historical evolution, posture assessment has advanced remarkably in terms of measurement precision, geometric reconstruction, and automated analysis [24,26,90,96,99,100]. Contemporary technologies can quantify selected anatomical and surface relationships with increasing precision, generate detailed three-dimensional representations of external body morphology, and identify complex postural patterns using AI-assisted techniques [22,23,49,96,98].

Paradoxically, these technological advances have not been matched by comparable improvements in the interpretation of postural data. While posture can now be measured, reconstructed, and classified with unprecedented sophistication, fundamental questions regarding its developmental meaning often remain unanswered [1,24,31,39,40,101,102].

This discrepancy may reflect a measurement–interpretation gap in the existing literature. Supplementary Table S3 summarizes how the principal scientific questions changed across successive methodological approaches and which interpretative limitations remained unresolved. Technological progress has largely focused on improving the ability to detect, describe, and quantify the postural characteristics of the body. In contrast, comparatively less attention has been directed toward understanding how postural patterns emerge, evolve, and interact with growth, maturation, and biological variabilities [1,2,24,39,40,103].

### 8.2. The Persistent Problem of Normality

One manifestation of the measurement–interpretation gap is the absence of a universally accepted definition of a normal posture [31,104]. Across ages and populations, classification systems, quantitative indices, imaging methods, and computational approaches have not produced a single consensus [10,84,89,101,102]. This difficulty is consistent with the biological variability in growth, body proportions, maturation, neuromuscular organization, and environmental adaptation [1,39,40]. Developmental and anthropological traditions recognized this variability long before modern imaging and AI, suggesting that developmental distributions and trajectories may provide more informative reference frameworks than a single ideal postural configuration.

### 8.3. From Static Configurations to Developmental Phenotypes

A common feature of many historical and contemporary approaches is their focus on postural configuration at a single time point rather than on longitudinal change across repeated observations [5,7,8,18,22,23,26,44,49,85,86,89,93,94,96,105]. Posture is frequently evaluated as a configuration observed at a particular point in time, whether assessed through visual observation or through anthropometric measurements [76,101], three-dimensional reconstruction [33,57,90,106], or artificial intelligence-assisted analyses [24,91]. These approaches provide valuable information regarding current morphology but offer limited insight into developmental trajectories.

However, from a developmental perspective, repeated observations of postural configuration may be interpreted collectively as a developmental phenotype characterized by individual patterns of change during growth and maturation. Individual postural characteristics emerge through continuous interactions among biological development, neuromuscular adaptation, environmental exposure, physical activity, and behavioral factors [1,31,39,40,107,108].

Consequently, understanding postural development requires moving beyond isolated descriptions toward frameworks that incorporate temporal change and developmental context [1,31,39,40,107].

### 8.4. The Need for a New Conceptual Framework

The historical evolution of posture assessment demonstrates that increasingly sophisticated methods have progressively improved the ability to observe, classify, measure, reconstruct, and analyze postural patterns in recent years. However, the interpretation of these patterns remains comparatively underdeveloped [24,36,109]. This observation does not diminish the importance of technological advancement. Rather, it suggests that future advances may depend less on further improvements in measurement accuracy and more on the development of conceptual frameworks that integrate biological variability, longitudinal observations, developmental trajectories, and predictive modeling [31,107,110]. Such considerations provide the rationale for the proposed concept of Developmental Postural Modeling, which seeks to extend posture assessment beyond pattern recognition toward longitudinal interpretation of postural development.

## 9. Developmental Postural Modeling

### 9.1. Why Developmental Modeling?

The historical evolution of posture assessment has revealed a consistent pattern. Successive methodological advances have progressively improved the ability to observe, classify, measure, reconstruct, and analyze postural characteristics of the human body. [18,22,24,36,111]. Nevertheless, posture continues to be interpreted predominantly in terms of anatomical configurations observed at a specific point in time [1,39,40].

This limitation may reflect a mismatch between predominantly cross-sectional assessment methods and the longitudinal nature of postural development. A clinical posture assessment describes body configuration at a defined time point, whereas postural development concerns changes in postural characteristics over time. During childhood and adolescence, these changes occur within the biological processes of growth and maturation. Consequently, approaches designed primarily to describe current morphology provide only partial information about postural development. From this perspective, the next major challenge in posture research may not be acquiring increasingly precise measurements but developing conceptual frameworks that can interpret postural changes over time.

### 9.2. Posture as a Developmental Phenotype

Developmental postural modeling begins with a conceptual assumption that differs from that of most traditional assessment approaches. Rather than interpreting an isolated postural configuration as a complete representation of an individual, DPM considers repeated postural observations within a developmental phenotype shaped by skeletal growth, biological maturation, neuromuscular adaptation, physical activity, environmental exposure, and behavioral influences [1,39,40,70].

Within this framework, posture remains an observable configuration at a defined time point, whereas postural development refers to longitudinal change in postural characteristics. Individual observations are therefore interpreted in relation to developmental context, temporal change, and trajectory rather than solely from their cross-sectional appearance.

This perspective is consistent with concepts previously emphasized in anthropological, auxological, and developmental traditions [1,39,40,41], while contemporary imaging, computational analysis, and AI provide new methodological opportunities for investigating these concepts [22,49,96]. It shifts the primary focus from describing postural morphology at a single time point to understanding how postural characteristics change across growth and maturation.

### 9.3. Developmental Trajectories and Longitudinal Observation

A central component of developmental modeling is the analysis of changes over time. Historically, postural assessments have predominantly relied on cross-sectional observation. Although such assessments provide valuable information regarding current body alignment, they offer limited insights into developmental dynamics. Two individuals presenting with similar postural characteristics at a given age may subsequently follow markedly different developmental pathways [1,39,40]. Longitudinal observation enables repeated postural configurations to be evaluated collectively as a developmental trajectory. Repeated assessments create opportunities to identify patterns of progression, stabilization, adaptation, compensation, and recovery. These trajectories may ultimately prove to be more informative than isolated measurements obtained at a single time point. From this perspective, the primary object of longitudinal analysis shifts from an isolated postural configuration to change in postural characteristics across repeated observations.

### 9.4. Digital Phenotyping and Artificial Intelligence

Recent advances in three-dimensional imaging, wearable technologies, motion-analysis systems, and artificial intelligence have expanded the range and dimensionality of data potentially available for longitudinal posture research [22,96,112].

Rather than relying on isolated measurements, developmental modeling may use multidimensional datasets that integrate geometric, functional, biomechanical, behavioral, and environmental data. These data can be used to characterize individual developmental profiles and identify recurring developmental patterns across populations. Within DPM, repeated digitally acquired observations of postural configuration are proposed as potential components of longitudinal digital phenotypes. This remains a research proposition rather than an established application.

Artificial intelligence may serve as an important enabling technology within this conceptual framework. Beyond current applications in detection, classification, segmentation, and measurement, future systems may support longitudinal analyses, trajectory identification, pattern discovery, predictive modeling, and individualized developmental assessments [93,96]. Importantly, artificial intelligence should not be regarded as a replacement for developmental interpretation but as a tool capable of managing the complexity of large multidimensional datasets.

### 9.5. Conceptual Framework for Developmental Postural Modeling

Developmental postural modeling may be conceptualized as the integration of five complementary domains:

- Morphology: Geometric and anatomical characteristics of posture;
- Function: Neuromuscular control, movement, and biomechanical performance;
- Development: Growth, maturation, and age-related change;
- Environment: Behavioral, social, and physical influences;
- Time: Longitudinal trajectories and temporal dynamics.

**Figure 3:**
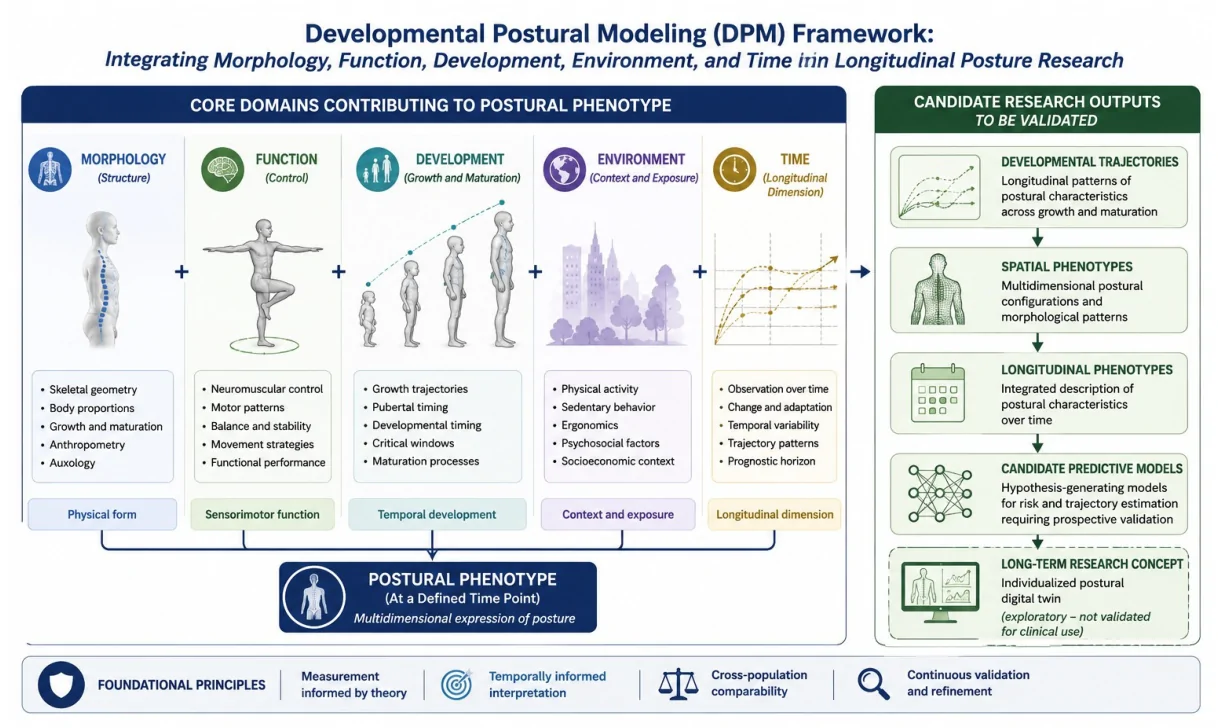
Developmental Postural Modeling Conceptual Framework: Integrating Morphology, Function, Development, Environment, and Time. Developmental Postural Modeling (DPM) framework. The framework integrates five core domains—morphology, function, development, environment, and time—to contextualize postural phenotypes observed at defined time points and investigate their longitudinal change. The right-hand panel illustrates candidate research outputs that require prospective validation. DPM represents a conceptual framework for future research and is not a validated clinical tool. The graphical rendering was produced with generative AI assistance based on the conceptual design and content specified by the authors.

Together, these domains provide a basis for interpreting an observed postural configuration within the dynamic processes of growth, maturation, function, environmental exposure, and time (Figure 3). In the present review, this framework is proposed primarily for research on children and adolescents and requires prospective validation before any clinical application.

### 9.6. Future Directions

Priority research needs include prospective longitudinal cohorts, developmental reference trajectories, standardized noninvasive imaging, and computational methods for trajectory and phenotype analyses. The DPM should be treated as a hypothesis-generating framework rather than a clinical tool. Whether it can improve prediction, risk stratification, or individualized management must be empirically tested. Individualized postural digital twins remain a speculative long-term possibility rather than being a current application.

## 10. Discussion

### 10.1. The Historical Paradox of Posture Assessment

This review demonstrates that while the past two centuries have yielded remarkable methodological and technological advances in posture assessment, they have simultaneously introduced profound epistemological paradoxes. Successive generations of researchers have successfully transitioned from subjective visual inspection to high-dimensional spatial reconstructions and automated machine learning-assisted pattern recognition [6,7,22,39,40,93,96]. However, these substantial advances in measurement and analytical capability [22,23,93,96] has not been matched by a corresponding evolution in interpretive, development-oriented frameworks [1,39,40,41]. Successive methodological approaches have resolved important technical challenges, such as minimizing observer dependency, reducing measurement error, and automating landmark extraction; however, they have predominantly evaluated postural configuration at isolated time points rather than examining longitudinal change within the biological processes of growth and maturation. Consequently, contemporary imaging systems can characterize external body-surface geometry with high spatial precision under controlled conditions; however, measurement precision should not be equated with criterion validity for underlying skeletal anatomy or with developmental and clinical interpretability. From an epistemological standpoint, the persistent measurement–interpretation gap reflects a historical trend in which technological capabilities have outpaced our conceptual capacity to interpret observed postural configurations within the dynamic, multifaceted processes of human growth and maturation.

### 10.2. Revisiting Developmental Traditions in the Era of Artificial Intelligence

An important observation emerging from this review is that several concepts emphasized by anthropological, auxological, and developmental traditions remain highly relevant despite profound technological advancements.

Researchers such as Wolański, Przewęda, Zeyland-Malawka, and other representatives of developmental approaches have consistently emphasized biological variability, age-dependent change, population diversity, and the limitations of universal normative standards [1,39,40,70]. Although these ideas were developed long before the emergence of digital imaging and artificial intelligence, many of the questions they raised remain unanswered today.

Interestingly, modern computational methods are increasingly capable of addressing questions that earlier developmental traditions identified as important but could only investigate using limited observational and measurement tools. Large-scale longitudinal datasets, three-dimensional imaging systems, and machine learning algorithms create opportunities to investigate developmental variability, identify postural phenotypes, and analyze developmental trajectories at scales previously unattainable. In this sense, contemporary technologies may provide tools capable of revisiting longstanding developmental questions, rather than replacing them.

Consequently, developmental and computational approaches may not be viewed as competing paradigms but as complementary components of a future framework for understanding postural development.

### 10.3. From Measurement Accuracy to Developmental Meaning

For pediatricians, orthopedic surgeons, and rehabilitation specialists, the distinction between measurement accuracy and developmental interpretation has potential clinical relevance; however, DPM itself is not yet available for clinical decision-making. During growth, a key challenge is distinguishing transient physiological variations from persistent or progressive structural changes. Cross-sectional measurements alone may be insufficient for this purpose, particularly when growth velocity and biological maturation differ substantially among children of the same chronological age.

If validated prospectively, trajectory-based approaches could eventually allow postural measurements to be interpreted relative to biological age, growth velocity, and maturation, rather than against rigid single-time-point thresholds. Such an approach might complement—not replace—established clinical guidelines and diagnostic pathways. Whether DPM can improve risk stratification, timing of follow-up, or personalization of rehabilitation remains a testable hypothesis requiring longitudinal validation.

### 10.4. Implications for Medical Imaging and Artificial Intelligence

The evolution of posture assessment closely parallels the broader developments in medical imaging and computational medicine. The transition from visual observation to digital reconstruction has transformed the way postural configuration is represented and analyzed, increasingly enabling image-based and three-dimensional characterization [22,23]. In contrast, contemporary artificial intelligence approaches have enabled the automated analysis of complex geometric datasets [96].

Current applications remain predominantly focused on detection, classification, segmentation, and measurement. However, the growing availability of longitudinal imaging datasets may allow future systems to move beyond static pattern recognition toward developmental trajectory analyses and predictive modeling [93,96].

Longitudinal studies have demonstrated that measurable postural characteristics change substantially during growth and maturation. For example, Widhe observed significant changes in thoracic kyphosis and lumbar lordosis between childhood and adolescence in the same individuals [32]. Contemporary digital imaging and three-dimensional surface-assessment methods now make it possible to acquire substantially richer representations of postural configuration at successive time points [33,107]. Building on these developments, repeated digitally acquired observations could eventually support longitudinal digital phenotyping of postural development; however, this remains a research proposition requiring prospective validation.

Three-dimensional reconstruction technologies, structured-light systems, surface topography, and point-cloud-based representations generate substantially richer information than that currently used in clinical practice. Much of this spatial information remains compressed into a limited set of derived parameters, potentially obscuring developmental and geometric relationships not readily captured by conventional measurements.

Therefore, future advances may depend not only on more sophisticated algorithms but also on conceptual frameworks that can preserve and interpret the multidimensional information already contained in modern imaging datasets. Rather than reducing detailed representations of body morphology and postural configuration to a limited set of summary indices, future approaches may benefit from characterizing multidimensional spatial phenotypes and interpreting their longitudinal change within a broader developmental context. Such developments could facilitate developmental phenotyping and trajectory-based assessment by linking repeated observations of postural configuration to longitudinal patterns of change during growth and maturation.

### 10.5. Limitations of Developmental Postural Modeling

Developmental Postural Modeling remains a conceptual and hypothesis-generating framework and has not yet been validated using prospective longitudinal data. Importantly, DPM does not redefine posture itself as a biological process. A posture assessment describes body configuration under defined conditions at a particular time point, whereas postural development refers to changes in postural characteristics across successive observations. The proposed framework therefore concerns the developmental interpretation of repeated postural observations rather than reinterpreting a single postural configuration as a dynamic process.

Several methodological requirements must be addressed before this framework can be empirically tested. These include standardized acquisition and imaging protocols, reproducible definitions of postural variables, sufficiently dense longitudinal datasets, appropriate consideration of biological maturation, and analytical methods that can distinguish measurement variability from genuine developmental change. Developmental trajectories would need to be derived from repeated observations within individuals rather than inferred from cross-sectional differences between age groups. Normative developmental models would also require sufficiently large, representative populations to characterize expected variability by age, sex, maturation, and other relevant biological or environmental factors.

The framework does not currently specify which morphological, functional, developmental, or environmental variables provide the greatest information for trajectory modeling, nor does it establish validated thresholds for distinguishing expected developmental variability from clinically relevant deviation. Similarly, predictive modeling, risk stratification, and individualized forecasting remain research objectives rather than demonstrated DPM capabilities. Any proposed relationship between a developmental trajectory and subsequent musculoskeletal outcome would require prospective external validation before clinical interpretation.

Accordingly, DPM should currently be regarded as a conceptual framework and research agenda for linking repeated observations of postural configuration with developmental context and longitudinal change. Its potential clinical utility, generalizability, and predictive value remain to be established empirically, initially in children and adolescents, in whom growth and maturation provide the clearest biological context for investigating developmental trajectories.

### 10.6. Strengths and Limitations of This Review

A principal strength of this review is its integration of scientific traditions that are usually considered separately. Historical orthopaedic concepts, anthropology and auxology, postural classification, quantitative measurement, three-dimensional imaging, and artificial intelligence-assisted analysis are examined within a common conceptual framework. This broad perspective makes it possible to distinguish advances in the acquisition and representation of postural configuration from the separate challenge of interpreting how postural characteristics change during growth and maturation. The review also brings greater international visibility to developmental concepts originating from the Polish anthropological, auxological, orthopaedic, and physical-education traditions, which have received comparatively limited attention in the international posture literature.

Several limitations arise from the historical and conceptual narrative design. The review was intended to identify major conceptual and methodological transitions rather than to provide exhaustive retrieval or quantitative synthesis of all available evidence. Formal risk-of-bias assessment was therefore not performed. The literature spans more than two centuries, encompasses heterogeneous scientific traditions, and uses terminology whose meaning has changed substantially over time. Historical sources are also unevenly indexed, and several influential publications are available only in Polish or other non-English languages. These characteristics necessarily require interpretative judgment when relating historical concepts to contemporary terminology.

The literature search was iterative and domain-based, and a complete prospectively archived retrieval log was not available for the original review process. Consequently, the present review should not be interpreted as a systematic review, and the absence of formal quantitative study-selection denominators limits complete reproducibility of the original historical search. To improve methodological transparency, the search domains, source-selection principles, eligibility and exclusion criteria, final search date, and representative database-specific search strategies are reported. These elements should not be interpreted as a retrospective reconstruction of a systematic-review search process.

Finally, the proposed historical sequence and DPM framework constitute an interpretative synthesis derived from the reviewed literature rather than an empirically validated taxonomy. The distinction between posture as a time-specific observable configuration, postural control as a sensorimotor process, and postural development as longitudinal change is therefore central to the interpretation advanced in this review. Prospective longitudinal research will be required to determine whether repeated posture assessments can reliably define developmental trajectories and whether such trajectories provide information beyond established cross-sectional measurements.

## 11. Conclusions

The history of posture assessment reflects a progression from descriptive observation and typological classification to quantitative measurement, three-dimensional reconstruction, and artificial intelligence-assisted analysis. These traditions have accumulated rather than simply replacing one another, progressively expanding the ability to describe, quantify, and analyze postural configuration.

Despite these advances, several fundamental challenges remain unresolved. There is no universally accepted definition of normal posture, longitudinal developmental evidence remains limited, and the clinical and prognostic significance of many postural characteristics is uncertain. A posture assessment describes body configuration under defined conditions at a particular time point; postural development, in contrast, concerns changes in postural characteristics across time. The unresolved challenge is therefore not simply how accurately a configuration can be measured, but how repeated observations should be interpreted within growth and maturation.

A central finding of this historical synthesis is the persistence of a measurement–interpretation gap. Increasingly sophisticated measurement and imaging technologies provide detailed representations of external morphology and postural geometry, while artificial intelligence extends the capacity for automated feature extraction, classification, and pattern recognition. However, greater technical precision does not by itself establish developmental significance, normative meaning, prognosis, or future trajectory.

Developmental Postural Modeling is proposed as a conceptual framework for addressing this gap. It does not redefine posture as a dynamic process and is not intended to replace existing classification systems, quantitative measurements, three-dimensional imaging, or AI-assisted analysis. Rather, DPM proposes that repeated observations of postural configuration be interpreted together with morphology, function, growth and maturation, environmental context, and time to investigate longitudinal developmental trajectories.

At present, DPM should be regarded as a hypothesis-generating research framework rather than a validated diagnostic, prognostic, or treatment-planning method. Its initial application is most strongly justified in children and adolescents, in whom growth and maturation provide an explicit developmental context. Prospective longitudinal studies are required to determine whether developmental trajectories can be reproducibly identified, whether normative trajectory models can be established, and whether such information provides clinically meaningful value beyond conventional cross-sectional assessment.

Future progress in posture research may therefore depend less on measuring individual postural configurations with ever greater precision and more on understanding the developmental trajectories revealed by repeated observations over time.

## Figures and Tables

**Table 1:** Historical Evolution of Human Posture Assessment and Its Conceptual Focus.

Era	Approximate Period	Dominant Approach	Primary Object of Assessment	Typical Methods	Main Question
Anthropological Foundations	1741–1900	Descriptive observation	Whole individual	Clinical observation, anthropometry [6]	What constitutes normal posture?
Classification Era	1900–1960	Typological classification	Posture type	Staffel [7], Brown [8], Dega [38], Wolański [39,40], Zeyland-Malawka [41] classifications	Which posture category best describes an individual’s posture?
Quantification Era	1950–1990	Measurement-based assessment	Selected anatomical parameters	Plumb line [5], Debrunner kyphometer [42], inclinometry [43], radiography [44]	How can posture be measured objectively?
Surface Assessment Era	1980–2010	Non-invasive geometric assessment	Back surface morphology	Moiré topography [16,45], photogrammetry [12,46,47,48], rasterstereography [22,49]	Can external body geometry reflect spinal alignment?
Three-Dimensional Reconstruction Era	2000–present	Spatial reconstruction	Three-dimensional trunk geometry	Surface topography [17,18,19,50,51,52,53], RGB-D systems [34], DIERS Formetric [54,55,56,57], Structured light [58,59,60,61,62,63]	How can posture be represented in three-dimensional space?
Artificial Intelligence Era	2015–present	Automated interpretation	Complex postural datasets	Machine learning, computer vision, markerless pose estimation [24,64,65]	Can algorithms recognize clinically meaningful postural patterns
Developmental Modeling Era (proposed)	Emerging	Longitudinal developmental modeling	Longitudinal change in postural characteristics	Longitudinal monitoring, trajectory analysis, multidimensional computational analysis, and digital phenotyping [Concept proposed in the present review.]	How do postural characteristics change across development, and can repeated observations define reproducible developmental trajectories?

## Data Availability

No new data were created or analyzed in this study.

## Supplementary Materials

The following supporting information can be downloaded at https://www.mdpi.com/article/10.3390/jcm15187197/s1, Table S1: Representative Database-Specific Search Strategies for the Principal Thematic Domains of the Narrative Review; Table S2: Evolution of Posture Assessment Technologies and Conceptual Frameworks; Table S3: Evolution of the Scientific and Clinical Questions Shaping Successive Posture Assessment Approaches.
