# Supplementary material for: Developmental Postural Modeling: A Historical and Conceptual Narrative Review of Posture Assessment
Source: J Clin Med. 2026 Sep 16;15(18):7197. doi: 10.3390/jcm15187197 (PMC13607638; doi:10.3390/jcm15187197)
Supplement: Supplementary file 1 [file jcm-15-07197-s001.zip › jcm-4420891-supplementary.pdf]

Supplementary Table S1. Representative Database-Specific Search Strategies for the Principal Thematic Domains of the Narrative Review.

The literature search was iterative and domain-based rather than based on a prospectively registered single search strategy. The strategies below document representative searches used for the principal thematic domains and should not be interpreted as verbatim reconstructions of every search iteration. The final literature search was completed on 30 May 2026.

Source	Role in Review	Representative Search Strategy	Search Field / Mode	Restrictions / Notes
PubMed/MEDLINE	Principal bibliographic database	("posture" OR "postural assessment" OR "body posture") AND (development* OR growth OR maturation OR auxology OR anthropometry)	Database search using representative query; PubMed automatic term mapping as applicable	No publication-date or document-type restriction was applied; final search completed 30 May 2026. The primary evidence base was English-language literature; selected non-English historical/conceptual sources were considered as described in Methods.
Scopus	Principal bibliographic database	TITLE-ABS-KEY((posture OR "postural assessment") AND (photogrammetry OR "surface topography" OR rasterstereography OR "structured light" OR "3D reconstruction"))	TITLE-ABS-KEY	No publication-date or document-type restriction was applied; final search completed 30 May 2026.
Web of Science	Principal bibliographic database	TS=((posture OR "postural assessment") AND ("artificial intelligence" OR "machine learning" OR "computer vision" OR "pose estimation"))	Topic (TS)	No publication-date or document-type restriction was applied; final search completed 30 May 2026.
Google Scholar	Supplementary discovery source	"posture assessment" development growth maturation; "posture classification" Wolański; "surface topography" posture scoliosis; AI posture assessment	Relevance-based supplementary discovery	Used for relevance-based citation tracking, historical-source discovery, and retrieval of sources not readily identifiable through conventional indexing. No fixed numerical result limit was prospectively applied; results were not used to generate formal retrieval denominators.
Semantic Scholar	Supplementary discovery source	posture assessment developmental trajectories; 3D surface topography posture; artificial intelligence posture assessment	Relevance-based supplementary discovery	Used for relevance-based citation tracking and interdisciplinary discovery. No fixed numerical result limit was prospectively applied; results were not used to generate formal retrieval denominators.

Supplementary Table S2. Evolution of Posture Assessment Technologies and Conceptual Frameworks.

The table summarizes major assessment approaches according to their primary object, measurement principle, output, dimensionality, spatial representation, strengths, limitations, and conceptual contribution. Developmental Postural Modeling (DPM) is presented as a proposed research framework rather than an established assessment method.

Approach / Method	Primary Object of Assessment	Measurement Principle	Output	Dimensionality	Spatial Representation Framework	Main Strengths	Main Limitations	Historical Significance / Conceptual Contribution
Visual Observation [6–8]	Global body alignment	Clinical inspection	Qualitative assessment	Observer-dependent	Human visual perception	Holistic assessment; no equipment required	Subjective; limited reproducibility	Introduced holistic pattern recognition based on simultaneous evaluation of alignment, symmetry, contours, and clinical signs.
Staffel Classification [7]	Sagittal postural patterns	Typological observation	Posture categories	2D descriptive	Sagittal body profile	Organized recurring postural patterns	Qualitative	One of the earliest systematic attempts to

Approach / Method	Primary Object of Assessment	Measurement Principle	Output	Dimensionality	Spatial Representation Framework	Main Strengths	Main Limitations	Historical Significance / Conceptual Contribution
								organize postural diversity into recognizable categories.
Brown Classification [8]	Global posture quality	Population grading system	Grades A-D	2D descriptive	Overall body alignment	Rapid large-scale screening	Limited anatomical detail	Demonstrated that posture assessment could be standardized for large populations.
Dega's Preventive Orthopaedic Approach [38]	Developmentally contextualized posture assessment	Clinical developmental evaluation	Developmental interpretation	2D descriptive	Functional body alignment	Preventive perspective	Limited quantification	Linked posture assessment with preventive orthopaedics and developmental interpretation.
Wolański Classification [39,40]	Developmental postural types	Typological developmental analysis	Kyphotic, balanced, lordotic types	2D descriptive	Sagittal body profile	Integration of posture and human biology	Predominantly cross-sectional	Shifted attention from posture categories alone toward developmental variability and biological maturation.
Zeyland-Malawka Classification [41]	Population-based developmental postural patterns	Simplified developmental classification	Practical posture categories	2D descriptive	Sagittal body profile	Applicable to large population studies	Qualitative	Adapted developmental concepts for practical clinical, educational, and epidemiological use.
Plumb Line Assessment [42]	Alignment relative to gravity	Reference to vertical axis	Alignment deviations	2D	Gravity-based vertical axis	Simple objective reference	Limited spatial information	Introduced posture assessment relative to gravitational loading and standing alignment.
Anthropometric Measurements [1]	Body proportions and alignment	Linear and angular measurements	Numerical parameters	2D	Anatomical landmarks	Quantification	Landmark-dependent	Initiated objective measurement of postural characteristics.
Cobb Angle (Radiography) [45]	Spinal deformity	Radiographic angle measurement	Cobb angle	2D	Standing anatomical projection	Standardized clinical metric	Projection-dependent; ionizing radiation	Became a clinical reference standard for quantifying spinal deformity under weight-bearing conditions.
Photography	External body appearance	Static image acquisition	2D image	2D	Image plane	Permanent documentation	Projection distortion	Transformed transient observation into reproducible visual documentation.
Photogrammetry [15,47–49]	Landmark geometry	Geometric image analysis	Distances, angles, ratios	2D / pseudo-3D	Landmark coordinate system	Quantitative image analysis	Limited depth representation	Enabled objective measurements to be extracted from photographic images.
Moiré Topography [16,46]	Surface contours	Optical fringe projection	Surface contour map	Surface representation	Surface height field	Non-invasive visualization	Limited anatomical specificity	Introduced body-surface morphology as an object of posture assessment.

Approach / Method	Primary Object of Assessment	Measurement Principle	Output	Dimensionality	Spatial Representation Framework	Main Strengths	Main Limitations	Historical Significance / Conceptual Contribution
Rasterstereography [22,50]	Trunk surface geometry	Raster projection and triangulation	3D surface model	3D	Cartesian XYZ coordinate system	Radiation-free 3D reconstruction	External surface only	Marked the transition from parameter measurement to three-dimensional geometric reconstruction.
Surface Topography [17–19,51–54]	Trunk morphology	Optical surface reconstruction	Surface geometry	3D	Cartesian XYZ coordinate system	Comprehensive trunk representation	No direct vertebral visualization	Shifted focus from individual measurements toward integrated geometric characterization of posture.
Structured-Light Systems [59–64]	Dense body geometry	Pattern projection and triangulation	Dense 3D reconstruction	3D	Dense XYZ point cloud	High geometric fidelity	External morphology only	Enabled digital geometric representation of the body as a spatial object rather than a set of measurements.
RGB-D Systems [34]	Spatial morphology	Depth sensing	Depth map and point cloud	3D	Depth-map / point-cloud space	Rapid acquisition	Sensor limitations	Facilitated routine generation of dense digital representations of posture.
Computer Vision [64,65]	Visual postural patterns	Automated feature extraction	Digital parameters	2D / 3D	Computational feature space	Automation	Dataset dependence	Reduced dependence on manual landmark identification and measurement.
Artificial Intelligence [24–26,65,66]	Complex geometric and image-derived patterns	Machine learning and pattern recognition	Classification, estimation, and pattern recognition	Multidimensional	High-dimensional feature space	Pattern discovery and automated analysis	Dataset dependence; interpretability and external-validation challenges	Shifted posture assessment from measurement toward automated recognition and modeling of complex patterns.
Developmental Postural Modeling (proposed)	Longitudinal change in postural characteristics	Integration of repeated postural observations with morphology, function, development, environment, and time	Candidate developmental trajectories and longitudinal phenotypes	Multidimensional	Longitudinal developmental space	Development-oriented interpretation of repeated observations	Conceptual framework requiring prospective longitudinal validation	Proposed framework linking repeated postural observations with developmental context and longitudinal change.

Supplementary Table S3. Evolution of the Scientific and Clinical Questions Shaping Successive Posture Assessment Approaches.

The table emphasizes the principal question addressed by each approach, the methodological response, the problem addressed, the residual limitation, and its legacy. The DPM row represents the authors' proposed research framework rather than an established clinical paradigm.

Era / Approach	What Was Assessed?	Scientific / Clinical Question Addressed	Main Innovation	What Problem Did It Address?	Main Limitation	Legacy for Future Developments
Andry (1741) [6]	Body alignment during growth	How can postural deformities be prevented during childhood development?	Preventive orthopaedic perspective	Linked posture with growth and prevention	No systematic assessment framework	Established posture as a developmental and preventive concern
Early Clinical Observation [6–8]	Global body alignment	How can abnormal posture be recognized clinically?	Structured visual examination	Enabled bedside assessment without equipment	Subjective	Introduced holistic pattern recognition
Staffel Classification [7]	Sagittal postural patterns	How can postural diversity be organized?	Typological classification	Created recognizable posture categories	Qualitative	Established posture classification as a scientific concept
Brown Classification [8]	Population posture grades	How can large populations be screened efficiently?	Standardized grading (A-D)	Enabled large-scale screening and comparison	Limited anatomical detail	Introduced population-based posture assessment
Adams Forward Bend Test [13,14]	Trunk asymmetry and rotation	How can scoliosis-related asymmetry be detected early?	Dynamic clinical screening test	Improved identification of rotational deformity	Screening rather than quantification	Remains a cornerstone of scoliosis screening
Dega's Developmental Approach [38]	Developmentally contextualized posture assessment	How can posture assessment support prevention and early intervention?	Preventive orthopaedic interpretation	Connected posture assessment with developmental clinical reasoning	Limited quantification	Reinforced developmental and preventive perspectives
Wolański Classification [39,40]	Developmental postural types	What is the role of biological development in postural variability?	Developmental interpretation	Shifted focus from posture type to developmental diversity	Predominantly cross-sectional	Introduced biological variability into posture assessment
Zeyland-Malawka Classification [41]	Population developmental postural patterns	How can developmental classifications be used in population studies?	Simplified developmental framework	Improved applicability in population studies	Qualitative	Facilitated large-scale developmental posture research
Plumb Line Assessment [42]	Alignment relative to gravity	How can posture be evaluated against an objective external reference?	Gravity-based reference axis	Reduced purely subjective assessment	Two-dimensional simplification	Introduced an external biomechanical reference
Anthropometric Assessment [1]	Body proportions and alignment	How can postural characteristics be quantified objectively?	Linear and angular measurements	Enabled numerical comparisons	Landmark-dependent	Initiated quantitative posture assessment
Cobb Angle [45]	Spinal deformity	How can spinal deformity be measured reproducibly?	Standardized radiographic metric	Enabled objective clinical monitoring	Projection-dependent; ionizing radiation	Became a clinical reference standard for spinal deformity
Photogrammetry [15,47–49]	Landmark geometry	How can objective measurements be extracted from images?	Geometric image analysis	Combined documentation with quantification	Limited depth information	Bridged visual assessment and quantitative analysis
Moiré Topography [16,46]	Surface morphology	How can body-surface shape be visualized non-invasively?	Optical surface mapping	Revealed surface asymmetries beyond simple measurements	Limited anatomical specificity	Introduced surface-based posture assessment
Rasterstereography [22,50]	Trunk surface geometry	How can postural configuration be reconstructed in three-dimensional space without radiation?	Optical 3D reconstruction	Enabled radiation-free geometric modeling	External surface only	Shifted posture assessment from measurement to reconstruction
Surface Topography [17–19,51–54]	Integrated trunk morphology	How can postural configuration be characterized as a geometric structure?	Whole-surface analysis	Captured complex spatial relationships	No direct vertebral visualization	Promoted geometric interpretation of posture
Structured-Light Systems [59–64]	Dense body geometry	How can complete external body geometry be captured digitally?	High-resolution 3D acquisition	Generated detailed digital body models	External morphology only	Enabled point-cloud-based posture analysis
RGB-D Systems [34]	Spatial morphology	How can postural configuration be digitized rapidly and inexpensively?	Depth sensing	Facilitated accessible 3D acquisition	Sensor limitations	Facilitated large-scale digital posture datasets
Computer Vision [64,65]	Visual postural patterns	How can postural features be extracted automatically?	Automated feature extraction	Reduced dependence on manual measurements	Dataset dependence	Established automated posture analysis
Artificial Intelligence [24–26,65,66]	Complex geometric and image-derived patterns	Can clinically relevant postural patterns be	Machine learning and	Identified relationships beyond	Dataset dependence,	Shifted emphasis from measurement toward automated

Era / Approach	What Was Assessed?	Scientific / Clinical Question Addressed	Main Innovation	What Problem Did It Address?	Main Limitation	Legacy for Future Developments
		recognized or estimated automatically?	multidimensional pattern analysis	predefined measurements	interpretability, and external validation	pattern recognition and modeling
Developmental Postural Modeling (proposed)	Longitudinal change in postural characteristics	How do postural characteristics change across growth and maturation, and can repeated observations define reproducible developmental trajectories?	Integration of morphology, function, development, environment, and time	Addresses the conceptual measurement-interpretation gap	Requires prospective longitudinal validation	Proposed framework for trajectory-based developmental research
